# The effects of musical feedback training on metacognition and self-directed learning

**DOI:** 10.3389/fnhum.2023.1304929

**Published:** 2023-12-18

**Authors:** Wen Li, Pravina Manoharan, Xuerong Cui, Fen Liu, Ke Liu, Lu Dai

**Affiliations:** ^1^School of the Arts, Universiti Sains Malaysia, Penang, Malaysia; ^2^School of Music Education, Zhejiang Conservatory of Music, Hangzhou, China; ^3^School of Primary Education, Fuzhou Preschool Education College, Fuzhou, China; ^4^Faculty of Education, Languages, Psychology and Music, SEGi University, Kuala Lumpur, Malaysia

**Keywords:** metacognition, metacognitive learning, musical feedback training, pre-service teachers, self-directed learning

## Abstract

**Introduction:**

Metacognition and self-directed learning are key components in educational research, recognized for their potential to enhance learning efficiency and problem-solving skills. This study explores the effects of musical feedback training on these competencies.

**Methods:**

The study involved 84 preservice teachers aged 18 to 21. Participants were randomly assigned to either an experimental group, which received musical feedback training, or a control group.

**Results:**

The findings indicate that musical feedback training effectively improved metacognitive abilities. However, its impact on the readiness for self-directed learning was inconclusive. A notable difference in metacognition and self-directed learning readiness was observed between the experimental and control groups during the session, indicating a significant interaction effect. Furthermore, a positive correlation was identified between metacognition and self-directed learning.

**Discussion:**

These results contribute to educational discourse by providing empirical evidence on the utility of musical feedback training in fostering metacognition. They also highlight the importance of consistent and long-term engagement in self-directed learning practices. The significance of these findings advocates for incorporating music feedback training into music education curricula to enhance metacognition and improve overall learning efficiency.

## 1 Introduction

The importance of metacognition and self-directed learning has become increasingly evident in music education as a result of the growing prevalence and promotion of online music education (Karatas and Arpaci, 2021; Khodaei et al., 2022; Marra et al., 2022). Metacognition is a concept that encompasses individual monitoring, self-evaluation, planning, and adjustment, which can them students manage their learning processes more efficiently (Ohtani and Hisasaka, 2018). Meanwhile, self-directed learning skills aid students in clarifying learning objectives and selecting strategies, especially with the reduced face-to-face interaction between teachers and students in online learning. Strengthening students’ metacognitive and self-directed learning abilities is a crucial educational task. This is because several studies (Bathgate et al., 2012; Power and Powell, 2018; Yokuş, 2021; Li et al., 2023) have found that the integration of metacognition and self-directed learning is indispensable for enhancing music learning efficiency. In accordance with that, various methods and strategies have been adopted in music education, including feedback training which is commonly used in metacognitive enhancement (Altıok et al., 2019; Molin et al., 2020; Karaoglan Yilmaz, 2022; Lee et al., 2023).

In music education, feedback training, including self-feedback, immediate feedback, and delayed feedback, is often employed to enhance learning efficiency. Self-feedback typically involves students using recording tools to record their practice, allowing for careful playback and self-evaluation to identify areas of improvement. On the other hand, immediate feedback occurs within individual practice sessions where, upon completing a section, teachers or recording software provide instantaneous evaluations and suggestions. Finally, delayed feedback pertains to the provision of feedback after students’ completion of a comprehensive performance or practice session, wherein teachers or peers subsequently provide summary evaluations (Wu et al., 2020; Blackwell et al., 2023).

Previous research indicates that feedback research in music education has primarily focused on skill and technique enhancement (Molin et al., 2020; Brooks, 2022; McPherson et al., 2022). However, this focus on specific skills does not encompass how musicians employ metacognitive strategies to enhance the efficiency of self-directed practice. Accomplished musicians utilize metacognition to reflect on and optimize their practice methods and their ability to independently accomplish complex practice tasks (Bathgate et al., 2012; Benton, 2013). In many instances, these musicians autonomously devise practice plans by managing their time, arranging different practice tasks, and effectively exercising self-control (Colombo and Antonietti, 2017). The fact that accomplished musicians not only possess highly developed metacognitive abilities, but they also excel in self-directed learning skills (Bucura, 2020; Vogel, 2020; Kosokabe et al., 2021). Whether music training can strengthen metacognition and impact the level and behaviors of self-directed learning remains unclear. Whether the achievements of musicians are a direct result of music training or an indirect is an area that has not received significant attention. Considering the uncertain effects of music feedback training in enhancing metacognition, this study plans to use feedback training methods in music education to improve students’ metacognition, with the aim to influence the enhancement of self-directed learning, and further explore the relationship between metacognition and self-directed learning in music practice.

This study contributes to the existing body of knowledge in the fields of music education and cognitive psychology. Firstly, it utilizes an innovative approach to explore the impact of music feedback training on metacognition and self-directed learning abilities in pre-service teachers, an area yet to be fully explored. Secondly, this study corroborates the significant positive correlation between metacognition and self-directed learning within the context of music education. The structure of this paper as following: the section 1 Introduction, section 2 presents literature review on related topics. Section 3 describes the methodology employed, and section 4 presents the research results. Section 5 discusses these results, summarizing the main contributions, limitations of the study, and suggestions for future research.

## 2 Literature review

The literature review is structured around three key themes: “Music Learning and Metacognition,” “Metacognition and Self-directed Learning,” and “Feedback Training Enhances Metacognition.” Following this, a theoretical framework is summarized, leading to the formulation of research hypotheses and questions.

### 2.1 Music learning and metacognition

Metacognition, first introduced by Flavell in 1979, is defined as “cognition about cognition” (Flavell, 1979). It is mainly divided into two components: metacognitive knowledge and metacognitive regulation. Metacognitive knowledge pertains to our awareness of our own and others’ thought processes, while metacognitive regulation focuses on monitoring and adjusting these cognitive processes. In-depth exploration of these metacognitive activities, including understanding of knowledge, planning, monitoring of processes, evaluation of outcomes, execution of adjustments, and selection of strategies, was carried out by Ohtani and Hisasaka (2018) and Craig et al. (2020). They proposed that these activities are crucial for effective learning.

In recent years, with educators emphasizing metacognition in the learning process, the connection between metacognition and music education has been increasingly acknowledged. Most studies have concentrated on how to apply metacognitive teaching strategies to enhance the efficacy of music instruction (Benton, 2013; Colombo and Antonietti, 2017; Bonnaire and González-Moreno, 2023). For instance, Colombo and Antonietti (2017) suggested that incorporating metacognitive strategies in music education is invaluable. Furthermore, Power and Powell (2018) argued that metacognition should not only be seen as an effective learning tool but also incorporated into formal educational curricula.

However, research on directly enhancing metacognition through music training remains limited. Nevertheless, the few studies available offer valuable insights. For example, Mieder and Bugos (2017) found that offering self-practice strategies in music could significantly elevate learners’ metacognitive regulation capabilities. Similarly, Yokuş (2021) discovered that providing specific learning strategies for music learners effectively enhances their metacognitive activities.

Although these findings provide some valuable guidance for music education, many unanswered questions remain. For instance, how is metacognitive training specifically implemented in music practice? Is it cultivated in a formal instructional environment or developed gradually during self-directed learning? As Colombo and Antonietti (2017) mentioned, this question remains unresolved. However, one fact is clear: during music self-practice, students extensively engage in monitoring, evaluating, and adjusting (Benton, 2013; Li et al., 2023)—activities central to nurturing metacognitive abilities. In sum, there exists a strong linkage between music learning and metacognition, warranting further exploration.

### 2.2 Metacognition and self-directed learning

Music learning heavily relies on self-directed learning (Concina, 2019; McPherson et al., 2019). Knowles (1975) provided a foundational definition of self-directed learning, outlining how learners proactively diagnose their learning needs, set objectives, identify resources, and implement learning strategies. Metacognition plays a pivotal role in self-directed learning (Mieder and Bugos, 2017), especially in intricate online learning environments, demanding learners to autonomously establish goals, efficiently manage their learning practice, and devise and execute learning plans.

Several studies have delved into the interplay between metacognition and self-directed learning. For example, Ghomi et al. (2016) and Breed and Bailey (2018) found a mutual enhancement between the two. Despite the extensive study and recognition of the relationship between metacognition and self-directed learning in general and online education, research in the realm of music education remains relatively scarce.

### 2.3 Feedback training enhances metacognition

Metacognition, acting as a self-regulatory mechanism for an individual’s cognitive processes and behaviors, relies to some extent on external feedback for optimization (Connor et al., 2019; Molin et al., 2020; Karaoglan Yilmaz, 2022). Feedback provides learners with information regarding their current understanding and skill level, enabling them to refine their learning strategies. Past research primarily concentrated on designing and implementing effective feedback mechanisms to amplify metacognitive abilities, including peer feedback, teacher feedback, and feedback through electronic or video tools (Altıok et al., 2019; Molin et al., 2020; Lee et al., 2023). Among these diverse feedback modalities, musical feedback remains a less explored yet potentially transformative avenue.

This study discusses whether musical feedback training can enhance metacognition from the following perspectives:

Self-monitoring and evaluation: A core component of metacognition is the ability to monitor and evaluate one’s own learning and thought processes. In musical training, students receive feedback and subsequently evaluate their performance against it. This process of reflecting on one’s performance through feedback, identifying areas for improvement, strengthens learners’ abilities for self-monitoring and evaluation (Fritz et al., 2020, Tang et al., 2022; Blackwell et al., 2023).

Planning and debugging practice strategies: Metacognition also encompasses planning and adjusting learning strategies. In music training, feedback is often accompanied by evaluations and suggestions, guiding learners to better practice and refine their techniques. It prompts them to think about how to improve their practice strategies in the next steps, thereby fostering their capacities for strategic planning and adjustment (McPherson et al., 2022).

**FIGURE 1:**
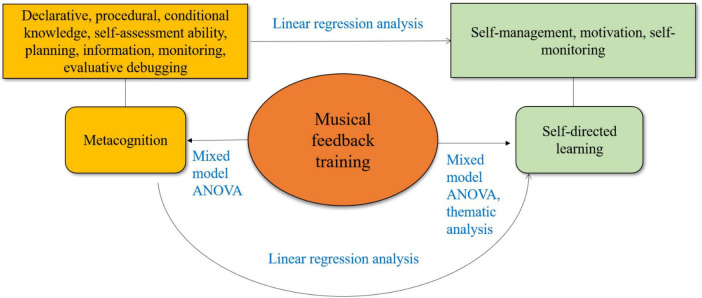
Theoretical framework.

The theoretical framework of this study is anchored in two key theories: Metacognition, originally introduced by Flavell (1979), and self-directed learning, as proposed by Knowles (1975). Metacognition plays a crucial role in the process of self-directed learning in music, assisting learners in planning, monitoring, and evaluating their learning strategies. Existing research predominantly focuses on the enhancement of musical skills, with less emphasis on how music training specifically influences metacognition and self-directed learning. This study utilized mixed model ANOVA and thematic analysis to explore how music feedback training impacts pre-service teachers’ metacognition and their readiness and behaviors in self-directed learning. Additionally, linear regression analysis was employed to discuss the influence of enhanced metacognition on self-directed learning. The theoretical framework of this study is shown in Figure 1.

### 2.4 Research hypotheses

H1. Music feedback training significantly improves participants’ metacognition.

H2. Pre-service teachers undergoing music feedback training exhibit significant improvements in their readiness and behavior for self-directed learning.

H3. A significant positive relationship exists between metacognition and self-directed learning among pre-service teacher in music practice.

### 2.5 Research questions

1. How does musical feedback training affect the improvement of metacognition?
2. Can musical feedback training effectively influence pre-service teachers’ readiness and behaviors in self-directed learning?
3. How does metacognition influence self-directed learning among pre-service teacher in music practice?

## 3 Methodology

To thoroughly understand the impact of musical feedback training on metacognition and self-directed learning, this study employed a mixed-method research design. Specifically, we integrated both quantitative survey questionnaires and qualitative thematic analysis. The research was conducted at a preschool education institute in China, targeting pre-service teachers who were randomly assigned into either the experimental group or the control group. The experimental group underwent specific singing training combined with music feedback, while the control group engaged in general singing lessons.

### 3.1 Participants

The participants comprised 84 pre-service teachers from a preschool education college in China, aged between 18 to 21 years. The average age of the experimental group was 19.43 years (SD = 0.496), whereas the control group’s average age was 19.47 years (SD = 0.629). Among all learners, 77.38% (*n* = 65) were female, and 22.62% (*n* = 19) were male.

#### 3.1.1 Sample size calculation

Utilizing the G*power software (Faul et al., 2009), the required sample size was calculated setting an alpha level of 0.05, a power of 0.8, and an anticipated medium effect size of 0.5. The computation suggested a minimum of 32 participants per group.

#### 3.1.2 Sample collection

Considering the study’s target demographic of pre-service teachers, a purposive sampling strategy was employed. Although initially intended to include 50 learners per group, certain participants failed to submit their weekly self-practice recordings and feedback cards on time, making it unclear whether they completed all training tasks. Thus, their data were excluded from the final analysis. Ultimately, data from 40 learners in the experimental group and 44 in the control group were collected, meeting the predetermined minimum sample requirement of 32 participants per group.

### 3.2 Research procedure

#### 3.2.1 Preliminary preparation and baseline testing

Prior to the experiment, all participants underwent a series of baseline assessments, evaluating both their intellectual and musical capabilities to ensure comparability between the experimental and control groups concerning prior knowledge and skill levels.

#### 3.2.2 Online teaching

Scheduled for an eight-week duration, all participants partook in an hour-long online session each week. All online educational activities were facilitated via the Tencent Meeting platform.

Distinguishing the experimental from the control group: Within the weekly one-hour online class, both groups delved into singing techniques for the initial 30 min, encompassing foundational singing knowledge, breathing techniques, proper standing posture, elementary vocal exercises, voice part determination, maintaining pitch accuracy, and linguistic expression techniques in singing. Subsequently, the experimental group spent the following 30 min mastering core feedback techniques and their practical application, guided and advised by an experienced tutor. Conversely, the control group, under the tutor’s guidance, continued with online singing technique training, excluding specific feedback training.

#### 3.2.3 Musical feedback training in singing practice (exclusive to the experimental group)

The rationale behind the experimental design is rooted in metacognition, which pertains to an individual’s thinking, awareness, and regulation of learning and cognitive processes. The core elements of metacognition include monitor, evaluation, reflection, and debugging strategies (Flavell, 1979; Ohtani and Hisasaka, 2018). Feedback training provides musicians with insights into their current understanding and skill levels, enabling them to monitor, evaluate, reflect, and debug their learning strategies and practice methods, thereby increasing metacognition.

Content and steps: During self-singing practice, learners recorded their renditions of each music piece. Immediately afterward, they compared their recordings with reference audio samples. During this phase, learners were encouraged to listen attentively, pinpoint discrepancies between their performance and the reference, thereby deriving both positive and negative feedback. Positive feedback reinforces learner confidence and motivation, while negative feedback requires reflection on the identified shortcomings.

The process entails:

1. Sound Recordings and **Monitoring**: Learners recorded during practice.
2. Immediate Playback: Post-completion of a musical phrase, the recording was played back instantly.
3. Compare Recordings and **Evaluate**: Comparisons were made with reference audio samples to extract positive or negative feedback.
4. **Reflection**: Continue with the same practice if positive feedback was received; reflect upon performance issues if negative feedback was encountered.
5. **Debugging** Practice Strategies: Participants need to debugging and improve their practice methods.

***Note:*** Selection of appropriate equipment: Ensuring the use of high-quality recording and listening equipment is essential. The use of professional recorders and high-quality headphones is recommended in order to accurately assess the quality of the sound and to identify errors in the exercises.

**FIGURE 2:**
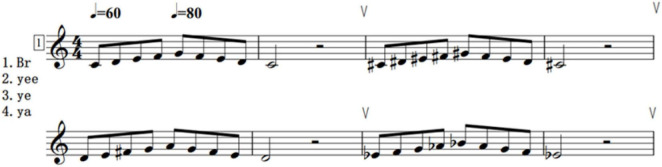
Vocal exercise routine.

Practice content planning: The training curriculum should incorporate vocal exercises. It is suggested that the chosen vocal exercises be divided into shorter musical phrases, which will facilitate a concentrated focus on monitoring, reflection, and adjustment. For instance, Figure 2 demonstrates a foundational scale training and employs basic vowels such as “Br (lip trill), Yee, Ye, Ya” for vocalization practice.

Recommendation on reference recording selection: When choosing a reference recording, it is advised to identify professional recordings that match the vocal range of the learner. The chosen sample should not only be suitable for the individual but also authoritative. For example, if the learner is a tenor, an exemplary tenor recording should be selected for comparison and imitation. Students should listen repetitively to establish and reinforce criteria for high-quality sound. During the early stages, emphasis should be placed on imitation exercises; thus, selecting a recording sample that matches one’s vocal characteristics is crucial.

#### 3.2.4 Post-class self-directed practice

Duration: A total of 8 weeks, practicing 3 times a week, with each session lasting 40 min.

Content for the self-directed practice includes:

1. Singing Feedback Training: Basic vocalization exercises, 25 min.
2. Breaking down and slowing down: 10 min. Break down a practice song into smaller parts or phrases; use slowing down to focus on the key parts of each phrase; try to work on more difficult parts alone, such as high notes or a particular technique.
3. Develop a personalized practice plan: 5 min. Based on the results of the day’s practice, identify strengths and weaknesses, and set goals and plans for the next practice session.

Difference between the experimental group and the control group: Experimental group required to use the singing feedback training method and submit a “Self-Practice Singing Reflection and Feedback Form” weekly.

Control group needs to ensure practice 3 times a week for 40 min each, with no specific method prescribed. Both groups are required to submit recordings of their self-practice weekly, allowing teachers to monitor their progress.

### 3.3 Instruments

#### 3.3.1 Musical learning metacognition questionnaire

To evaluate participants’ musical learning metacognition, this research utilizes the Musical Learning Metacognition Questionnaire (Li et al., 2023). This instrument, meticulously designed to reflect the distinct characteristics of music, builds upon the foundation of conventional metacognitive questionnaires. The development process of this questionnaire entailed several iterations of exploratory factor analysis (EFA), subsequently complemented by confirmatory factor analysis (CFA), which substantiated the validity of the factors identified. The resulting tool has exhibited robust reliability and validity in its application. The questionnaire adopts a 5-point Likert scale format and contains 35 items. The overall reliability (Cronbach’s α) of the questionnaire is 0.945. It’s divided into two major subscales: metacognitive knowledge and metacognitive regulation. Eight factors were identified: declarative knowledge (Cronbach’s α = 0.73), procedural knowledge (0.71), conditional knowledge (0.70), self-assessment of musical ability (0.61), planning (0.83), information management (0.76), monitoring (0.83), and evaluative debugging (0.81). These factors accounted for 66.67% of the total variance.

#### 3.3.2 Self-directed learning readiness scale

To measure participants’ self-directed learning capabilities, we employed the widely recognized Self-Directed Learning Readiness Scale (Fisher et al., 2001). This scale is not only trusted but has also passed structural validity tests. Although the original self-directed learning readiness scale consists of 44 items, for this research, we opted for 32 items. All items utilize a 5-point Likert scale. This tool, divided into three main dimensions: self-management, motivation, and self-monitoring, displays high internal consistency with Cronbach’s α values of 0.866, 0.863, and 0.855, respectively. Furthermore, it successfully underwent structural validity verification, further enhancing its reliability as an effective measurement tool.

#### 3.3.3 Self-practice singing reflection and feedback form

Inspired by Spafford and Haarhoff (2015), this reflection and feedback form was designed specifically to aid students in self-assessment and reflection during their singing learning process. The form includes the following prompts:

What long-term goals have you set for your singing practice?
This week, what specific singing exercises or techniques did you focus on?
What specific challenges or problems did you encounter in your singing practice?
What strategies or methods did you employ to address these challenges and strive to achieve your goals?
In this week’s learning, what areas do you feel need further improvement or strengthening?

### 3.4 Data collection and analysis method

At the beginning and end of the training, data on metacognition (based on the metacognition scale) and self-directed learning (using the self-directed learning readiness scale and the self-practice singing reflection and feedback form) were collected to assess the training’s impact. Before training started, baseline tests, including IQ (intelligence quotient) tests and musical ability assessments, were also conducted. After the training concluded, additional data from teacher and student interviews were collected.

#### 3.4.1 Quantitative analysis

This study uses the Mixed Model ANOVA (Boisgontier and Cheval, 2016) for the quantitative data analysis. This method enables us to investigate the simultaneous effects of the experimental and control groups (group, between-subjects factor) and the pretest and posttest sessions (session, within-subjects factor) on the dependent variables (metacognition and self-directed learning). It is particularly effective in revealing the training’s effectiveness and how it differentially influences the experimental and control groups across pretest and posttest periods.

#### 3.4.2 Qualitative analysis

Thematic analysis was employed to analyze the data gathered from the self-practice singing reflection and feedback form, along with the teacher and student interviews. This method excels in identifying and analyzing themes within the qualitative data, offering valuable insights into the study’s findings.

## 4 Results

Initially, a preliminary evaluation was conducted, assessing the initial differences between the two groups. An analysis of variance (ANOVA) was performed on pre-test scores for all tasks, using group (experimental vs. control) as the between-subjects factor. Moreover, no significant differences were detected between the two groups on the baseline assessments (IQ tests and musical ability evaluations). In this study, a *p*-value less than 0.05 is considered to indicate a significant difference.

To evaluate the training effects, a 2 (experimental vs. control) x 2 (pre-test vs. post-test) Mixed-Model ANOVA was adopted. In all statistical tests, the alpha level was set at 0.05. All group data underwent normality and homogeneity of variance tests, meeting the requirements for the Mixed-Model ANOVA. Descriptive statistics are presented in Table 1.

**TABLE 1:** Means (M) and standard deviations (SD) for metacognition and self-directed learning in both groups.

Construct	Control group (*n* = 44)		Experimental group (*n* = 40)	
	Pre-test	Post-test	Pre-test	Post-test
	M(SD)		M(SD)	
Metacognition	3.52 (0.45)	3.47 (0.37)	3.48 (0.44)	3.73 (0.41)
Metacognitive knowledge	3.48 (0.46)	3.54 (0.42)	3.54 (0.46)	3.80 (0.40)
Self-assessment of musical ability	3.52 (0.58)	3.45 (0.44)	3.55 (0.48)	3.66 (0.47)
Declarative knowledge	3.47 (0.59)	3.52 (0.65)	3.56 (0.60)	3.68 (0.46)
Procedural knowledge	3.42 (0.56)	3.64 (0.62)	3.49 (0.57)	3.88 (0.51)
Conditional knowledge	3.52 (0.59)	3.61 (0.61)	3.60 (0.60)	3.82 (0.58)
Metacognitive regulation	3.54 (0.48)	3.41 (0.38)	3.45 (0.46)	3.69 (0.45)
Planning	3.37 (0.63)	3.30 (0.56)	3.43 (0.56)	3.64 (0.57)
Information management	3.70 (0.55)	3.62 (0.48)	3.62 (0.48)	3.70 (0.55)
Monitoring	3.48 (0.59)	3.21 (0.43)	3.26 (0.64)	3.60 (0.52)
Evaluative debugging	3.60 (0.49)	3.48 (0.47)	3.49 (0.45)	3.63 (0.59)
Self-directed learning	3.60 (0.50)	3.50 (0.39)	3.55 (0.50)	3.73 (0.49)
Self-management	3.25 (0.59)	3.20 (0.55)	3.22 (0.57)	3.43 (0.66)
Motivation	3.82 (0.58)	3.73 (0.45)	3.76 (0.57)	3.88 (0.49)
Self-monitoring	3.64 (0.56)	3.48 (0.40)	3.57 (0.56)	3.73 (0.56)

### 4.1 Metacognition

**FIGURE 3:**
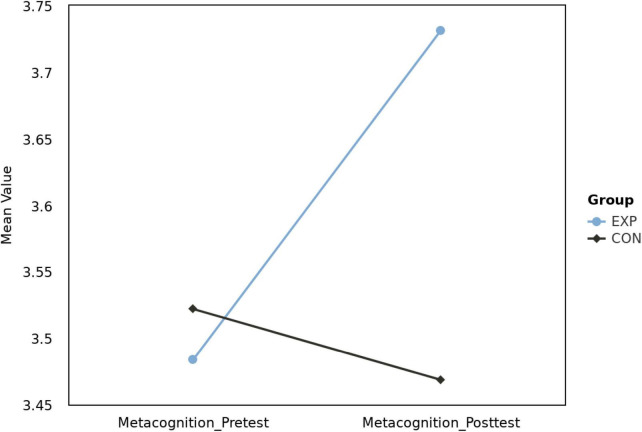
Mean metacognition scores: pre-test vs. post-test for experimental (EXP) and control (CON) groups.

The interaction effect revealed a significant difference in patterns of metacognitive scores between the experimental and control groups from pre-test to post-test, *F*(1, 82) = 6.33, *p* = 0.014, η_p_2 = 0.07. This suggests a different pattern of metacognitive development in the two groups before and after the musical feedback training. As illustrated in Figure 3, average metacognitive scores of the experimental (EXP) and control (CON) groups during pre-test and post-test were compared. *Post hoc* comparisons further showed that the experimental group’s metacognition scores were significantly lower at pre-test than post-test, *t*(82) = −2.86, *p* = 0.005, indicating a significant effect of the musical feedback training on metacognition. However, neither the main effects between the two groups nor those between pre and post-tests were significant.

#### 4.1.1 Metacognitive knowledge

The interaction effect for metacognitive knowledge was not significant. However, the experimental group’s post-test scores were significantly higher than their pre-test scores, *t*(82) = −2.78, *p* = 0.007. The main effect analysis revealed significant differences between groups and between pre-test and post-test scores for metacognitive knowledge. Additionally, values for the four dimensions included in the Metacognitive knowledge section were collected. These dimensions are: self-assessment of musical ability, declarative knowledge, procedural knowledge, and conditional knowledge. The study also computed the mean and standard deviation for each dimension in both pre-test and post-test phases, offering a clearer view of the variations across these dimensions. For detailed information, refer to Table 1.

#### 4.1.2 Metacognitive regulation

The interaction effect between the experimental and control groups for metacognitive regulation reached statistical significance, *F*(1, 82) = 9.02, *p* = 0.004, η_p_2 = 0.10. Further *post hoc* analysis revealed that the experimental group demonstrated a significant improvement in metacognitive regulation scores from pre-test to post-test, *t*(82) = −2.70, *p* = 0.008. The Metacognitive Regulation section encompasses four dimensions: planning, information management, monitoring, and evaluative debugging. The mean and standard deviation for these dimensions were calculated for both pre-test and post-test phases, providing a clearer depiction of the changes across these aspects. For specific data, please refer to Table 1.

### 4.2 Self-directed learning

For self-directed learning data, we first analyzed the data for self-directed learning readiness. Subsequently, reflection and feedback forms from all learners on self-directed practice were collected to analyze changes in students’ self-directed learning behaviors.

#### 4.2.1 Quantitative analysis of self-directed learning readiness

**FIGURE 4:**
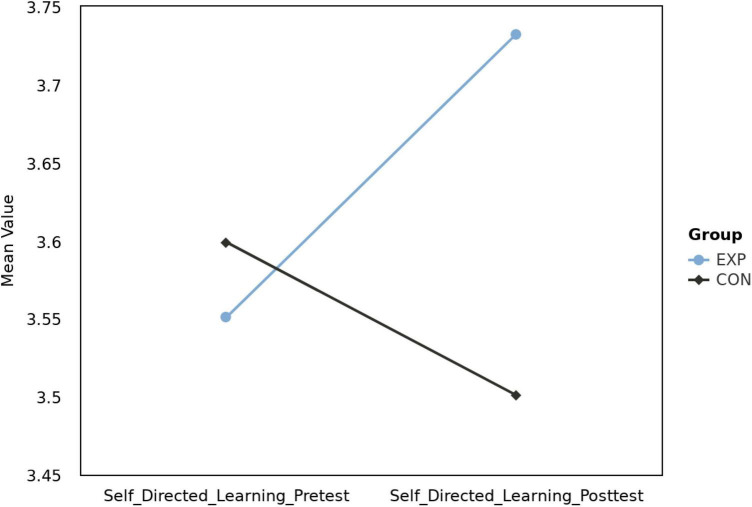
Mean self-directed learning readiness scores: pre-test vs. post-test for experimental (EXP) and control (CON) groups.

A significant interaction between “group” and “session” variables was observed, *F*(1, 82) = 4.20, *p* = 0.044, η_p_2 = 0.05. The significant interaction effect implies that the experimental and control groups differed in the pattern of scores on self-directed learning readiness during the pre-test and post-test. In other words, these two groups did not have the same trend in the level of self-directed learning readiness before and after the musical feedback training. As shown in Figure 4, we compared the average self-directed learning readiness scores of the experimental (EXP) and control (CON) groups during pre-test and post-test. This chart is intended to visually demonstrate the developmental trends of self-directed learning readiness in both groups before and after musical feedback training. However, in the *post-hoc* test for the experimental group, no significant differences were found, *t*(82) = −1.84, *p* = 0.070. This might imply that the experiment did not successfully induce changes within the experimental group in this aspect, or any changes were too subtle to manifest as statistically significant. The main effect for “session” was not significant, *F*(1, 82) = 0.37, *p* = 0.542, η_p_2 = 0.005. Similarly, the main effect for “group” was also non-significant, *F*(1, 82) = 1.43, *p* = 0.235, η_p_2 = 0.02.

Self-Directed Learning encompasses three dimensions: self-management, motivation, and self-monitoring. This study also calculated the changes in the mean values of these three dimensions before and after the training, as detailed in Table 1.

#### 4.2.2 Qualitative analysis of self-directed learning behaviors

Thematic analysis, a qualitative research technique, was employed to extract key themes from the data, providing deeper insights into the effectiveness of music feedback training on the behaviors associated with self-directed learning. We collated practice reflection and feedback forms submitted by students in the experimental group. Additionally, in-depth interviews were conducted with five students (three from the experimental group and two from the control group). These themes were primarily categorized into “Positive statements” and “Negative statements.” “Positive statements” were further broken down into five sub-themes, including enhanced self-management, increased self-monitoring, growth in learning motivation, and improvements in singing techniques.

Positive statements:

Enhancements in self-management: This includes setting clear goals and time planning.

“*I’ve set a clear practice plan, practicing for half an hour every Monday, Wednesday, and Friday after classes. Gradually, I’ve increased my vocal training time, progressing from 10 to 20 min. In the remaining 10 min, I practice songs I love.*”

Progress in self-monitoring:

“*Now, I use recording tools to regularly check the effectiveness of my practice to ensure they align with my goals.*”

Boost in learning motivation:

“*I understand that my practice sessions are relatively short and sometimes feel insufficient. But I’ve practiced using a piano app I downloaded on my phone. I really want to improve my singing skills. Even though my online learning sessions were short, they made me fall in love with singing.*”

Advancements in singing techniques:

“*Now, while singing, I don’t run out of breath halfway. I feel I can sing higher notes, all thanks to my teacher’s encouragement. I’ve consistently practiced as instructed by my teacher. I’ve realized more practice is needed, especially in aspects like breathing and transitioning to high notes.*”

Negative statements:

“*I still find it challenging to concentrate on self-practice. Using recording tools seems a bit difficult for me.*”

“*After training, I still feel there are many areas where I lack, such as adjusting my pitch and finding the right tune.*”

“*I’m not very clear about the concept of opening the throat. Even though some say my throat seems tense when I sing, I don’t know how to change that.*”

In conclusion, students have exhibited numerous positive changes in their behaviors related to self-directed learning. They’ve made progress not only in self-management, self-monitoring, and learning motivation. However, they still faced certain challenges, such as mastering some skills or having difficulty focusing.

### 4.3 Linear regression analysis

A linear regression analysis of pre-post differences was employed to evaluate the relationship between metacognition and self-directed learning readiness. Specifically, difference scores between pre-test and post-test were calculated for all participants, derived by subtracting the pre-test score from the post-test score. In this model, the difference score for metacognition served as the independent variable, while the difference score for self-directed learning readiness served as the dependent variable. Through the regression analysis, it was possible to more precisely assess how changes in metacognitive levels predict changes in self-directed learning readiness. It was also verified that the data met the basic assumptions of normal distribution and homoscedasticity, and given that only one independent variable was considered, multicollinearity was not an issue.

Results from the linear regression model showed a high fit, *F*(1, 82) = 108.41, *p* < 0.001, with an R^2^ of 0.57. This suggests that metacognition can explain nearly 57% of the variance in self-directed learning. The model further revealed that metacognition significantly predicted self-directed learning, specifically, *B* = 0.85, *t*(82) = 10.41, *p* < 0.001. In simpler terms, for every unit increase in metacognition, self-directed learning will increase, on average, by 0.85 units. Unstandardized regression equation: self-directed learning = −0.04 + 0.85*Metacognition. This underscores the potent predictive power of changes in metacognition for changes in self-directed learning.

**FIGURE 5:**
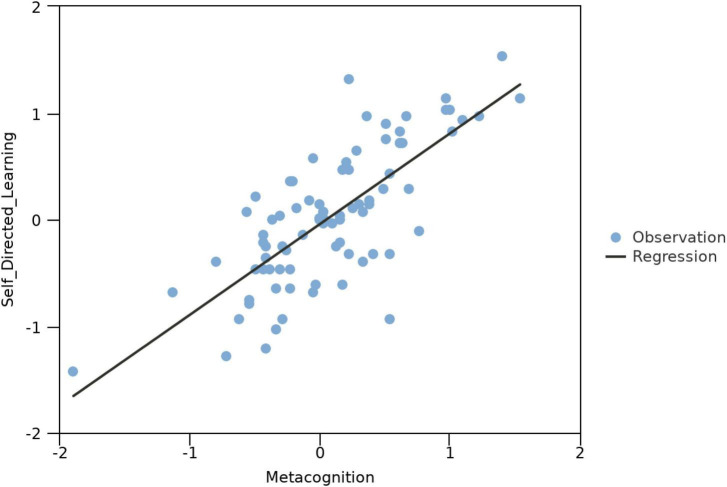
Scatter plot of the linear regression analysis between metacognition and self-directed learning.

The study further conducted regression analyses to explore the impact of various dimensions of metacognition (as independent variables) on the dimensions of self-directed learning (as dependent variables), utilizing pre and post-test data of all participants. The findings revealed correlation of significance between the declarative knowledge and evaluative debugging dimensions of metacognition and the self-management dimension of self-directed learning. Additionally, significant relationships were observed between declarative knowledge, planning, and monitoring with the motivation dimension, as well as between declarative knowledge, monitoring, and evaluative debugging with the self-control dimension. No significance was found between the other dimensions. Detailed results of the regression model can be referred to in Table 2. The linear relationship between metacognition and self-directed learning is visually represented in the scatter plot shown in Figure 5.

**TABLE 2:** Results for linear regression.

Regression analysis	*B*	SE	β	*t*	*p*
Metacognition → self-directed learning	0.85	0.08	0.75	10.41	<0.001
Declarative knowledge → self-management	0.18	0.08	0.18	2.34	0.021
Evaluative debugging → self-management	0.35	0.12	0.30	2.93	0.004
Declarative knowledge → motivation	0.18	0.06	0.20	2.89	0.004
Planning → motivation	0.22	0.08	0.25	2.94	0.004
Monitoring → motivation	0.17	0.08	0.19	2.10	0.037
Declarative knowledge → self-monitoring	0.24	0.07	0.27	3.66	<0.001
Monitoring → self-monitoring	0.19	0.09	0.21	2.20	0.030
Evaluative debugging → self-monitoring	0.26	0.10	0.25	2.52	0.013

### 4.4 Follow-up

As a subsequent step in research, interviews were conducted with three teachers involved in the music training to systematically uncover the strengths and weaknesses of the study. Inquiries centered on the teachers’ experiences with the singing feedback training, with a focus on aspects such as music training content, lesson planning, and the impact of online training on students. A qualitative analysis of this survey data was then undertaken. Teachers generally felt that the training effectively enhanced students’ metacognition and fostered habits of self-directed practice.

Positive statements:

*This module teaches students about self-directed learning and develops metacognitive and singing practice skills.*

*The greatest advantage of self-directed learning is that it helps students to complete their studies without teacher supervision.*

Negative statements:

*The training emphasizes singing content excessively, leading to insufficient coverage of metacognitive teaching content. As a result, my understanding of metacognition is not thorough.*

*Developing a habit of self-directed learning takes time. Some students did not submit their weekly practice recordings on time. The experimental design should consider robust supervisory management to ensure active participation from all students.*

## 5 Discussion

This research investigated the impact of musical feedback training on metacognition and self-directed learning for online learners. Specifically, it examined whether musical feedback training influences learners’ metacognition (RQ1) and self-directed learning (RQ2) and explored the relationship between metacognition and self-directed learning (RQ3).

### 5.1 Effect of Musical feedback training on Metacognition

For the first time, the study revealed that musical feedback training can enhance learners’ metacognition. A significant interaction effect on metacognition was observed, with a *p*-value of 0.014 and η_p_2 of 0.07. This indicates that the musical feedback training impacted the experimental group. To elucidate, the value of η_p_2 (0.07) suggests that the independent variable in this study explains 7% of the total variation in the dependent variable. Although this effect size falls within a small to medium range, given the complexity and diversity of factors in the online teaching environment, these findings remain practically significant. Moreover, post-tests for the experimental group revealed significantly higher scores than the pre-tests (*p* = 0.005), further affirming the potential value of musical feedback training in enhancing metacognition. These results align with earlier studies where electronic learning tools (Altıok et al., 2019; Connor et al., 2019; Molin et al., 2020; Lee et al., 2023) and feedback from peers and teachers (Krueger et al., 2017; Karaoglan Yilmaz, 2022) positively influenced metacognition. We also noted that while there was a significant interaction effect for subcomponents of metacognition, such as metacognitive regulation, there was no significant interaction for metacognitive knowledge. This is attributed to the immediate feedback from music training, aiding learners in effectively adjusting their learning behaviors or strategies. Since metacognitive regulation typically involves multiple dynamic aspects, such as planning, monitoring processes, and strategy adjustments (Müller and Seufert, 2018; Ohtani and Hisasaka, 2018; Craig et al., 2020; Li et al., 2023), musical feedback training exerts a more direct influence on it.

Given these findings, we recommend that music educators intensify their focus on feedback to nurture metacognition. Given the intricate and unpredictable nature of online contexts, teachers should explore innovative teaching methods or tools to specifically enhance students’ metacognitive performance.

### 5.2 Effect of musical feedback training on self-directed learning

The interaction effect on self-directed learning readiness was significant with a *p*-value of 0.044 and η_p_2 value of 0.05, indicating that the music training had differential impacts on the experimental and control groups—the former showing an increase and the latter a decline. This suggests the effectiveness of musical feedback training for the experimental group. However, post-test scores for this group were not significantly higher than the pre-tests (*p* = 0.070), indicating that the training did not induce a notable change in self-directed learning readiness. Literature suggests that changes in self-directed learning require prolonged influence and training (Robinson and Persky, 2020; Zhu et al., 2020) and are influenced by students’ early habits (Beckers et al., 2016; Onah et al., 2021). Given that this study spanned only 8 weeks, the duration might not have been sufficient to evoke a significant change in self-directed learning. Future studies could consider extending training duration to enhance the effectiveness of self-directed learning.

Qualitative analyses revealed that musical feedback training influenced students’ self-directed learning behaviors. Positive statements indicated improvements in self-management, self-monitoring, learning motivation, and singing techniques. For instance, students planned their practice times and effectively used technological tools to monitor their progress. However, some negative feedback emerged. Some students found it challenging to focus on self-practice, struggled with recording tools, and faced difficulties in adjusting pitch and relaxing their throats. Future course designs should particularly address and remedy these concerns.

### 5.3 Relationship between metacognition and self-directed learning

The study found that, within online music learning, metacognition indeed plays a crucial role in fostering the development of self-directed learning. Linear regression analysis revealed that changes in metacognition can explain 57% of changes in self-directed learning, and it holds a significant predictive power [*B* = 0.85, *t*(82) = 10.41, *p* < 0.001]. This implies that as metacognitive abilities increase, self-directed learning capacities correspondingly rise. This discovery resonates with findings from other educational researchers (Dagal and Bayindir, 2016; Jin and Ji, 2021; Khodaei et al., 2022), filling a research gap in the realm of online music education on metacognition and self-directed learning. Practically, this model suggests that educators should consider integrating more metacognitive training into their teaching practices. Future research can continue to delve into how this relationship is established and focus on enhancing self-directed learning capabilities by fostering metacognition among music students.

## 6 Conclusion

Empirical analysis has provided initial evidence that musical feedback training is efficacious in enhancing metacognitive levels, particularly with regard to its pronounced influence on metacognitive regulation. Our findings corroborate the close relationship between music training and metacognitive development. The study also highlighted the positive correlation between metacognition and self-directed learning in music education. With elevated metacognitive abilities, students can better identify, plan, monitor, and adjust their learning strategies, leading to more effective self-directed learning. However, in the online learning environment, without effective self-practice strategies, students’ readiness for self-directed learning tends to decline. In conclusion, metacognition, combined with musical feedback training, can act as a valuable asset in shaping self-directed learners, better equipped to navigate the intricacies of the online learning landscape. Future endeavors in this realm should continue to build upon these insights, providing comprehensive, actionable strategies for educators worldwide.

## 7 Limitations and suggestions for future research

Despite meticulous planning and design, several limitations of this study warrant attention in subsequent research. One limitation of this study is the reliance on qualitative research for follow-up measures, lacking quantitative data. Future research can consider incorporating a more comprehensive approach, integrating both qualitative and quantitative methods for a holistic examination of the issues. Secondly, the research was conducted with a limited sample size. Future studies could consider a larger scale sample to discern the effect of this approach and explore other strategies to improve pre-service teacher outcomes. Furthermore, the eight-week duration may not suffice for long-term training effects on self-directed learning. Extending the duration could enhance the effects of musical feedback training.

While this research primarily benefits the domain of music learning, broader implications exist for any pedagogical practice looking to foster metacognition and self-directed learning among students. Educators from diverse disciplines could potentially adapt musical feedback training tools and techniques to enrich their students’ learning experiences.

## Data availability statement

The raw data supporting the conclusions of this article will be made available by the authors, without undue reservation.

## Ethics statement

The studies involving humans were approved by the Human Research Ethics Committee, Universiti Sains Malaysia. The studies were conducted in accordance with the local legislation and institutional requirements. The participants provided their written informed consent to participate in this study.

## Author contributions

WL: Writing – original draft, Writing – review & editing. PM: Methodology, Supervision, Writing – review & editing. XC: Funding acquisition, Supervision, Writing – review & editing. FL: Project administration, Resources, Writing – review & editing. KL: Investigation, Resources, Writing – review & editing. LD: Investigation, Writing – review & editing.
